# Personal digital health hubs for multiple conditions

**DOI:** 10.2471/BLT.19.249136

**Published:** 2020-06-02

**Authors:** Mellick J Chehade, Lalit Yadav, Asangi Jayatilaka, Tiffany K Gill, Edward Palmer

**Affiliations:** a Discipline of Orthopaedics & Trauma, University of Adelaide, Level 5G 584, Royal Adelaide Hospital, Port Road Adelaide, South Australia, Australia 5000.; b School of Computer Science, University of Adelaide, Adelaide, Australia.; c Adelaide Medical School, University of Adelaide, Adelaide, Australia.; d School of Education, Faculty of Arts, University of Adelaide, Adelaide, Australia.

## Abstract

Multimorbidity is the presence of more than one chronic disease condition in an individual. Health-related, socioeconomic, cultural and environmental factors, as well as patient behaviour, all influence the outcomes of multimorbidity. Addressing these complex and often interacting biopsychosocial factors therefore requires a shift in treatment from a physical damage model towards person-centred integrated care with increased patient agency. Education influences behaviour and can be used to empower patients and their carers with greater agency, thus allowing greater responsibility for and control over the management of patient care. In this paper we reflect on our own learning as a community of health practitioners from different disciplines. Recognizing the increasing importance of patient agency in driving the evolution of health care, we describe the concept of a web-based personal digital health hub for integrated patient care. Informed by collaboration between patient, health and education communities, we share our early experience in the implementation of a health hub around a cohort of patients with hip fractures. We also describe a vision for future health care based on the co-creation of digital health hubs centred on patients’ and carers’ needs. The health hub could allow important advances and efficiencies to be achieved in workforce practice and education; patient and carer engagement in self-care; and the collection of patient-reported health data required for ongoing research and improvements in health care.

## Résumé

La multimorbidité est la présence de plus d'une maladie chronique chez un individu. L'aboutissement de la multimorbidité est influencé par des facteurs sanitaires, socio-économiques, culturels et environnementaux. Aborder ces facteurs biopsychosociaux complexes et souvent interdépendants requiert donc un changement de traitement, qui consiste à s'éloigner d'un modèle axé sur les dommages physiques pour se rapprocher d'un modèle de soins intégré et centré sur la personne, allié à une meilleure implication du patient. L'éducation a un impact sur le comportement et peut être utilisée pour renforcer la capacité d'agir des patients et de leurs soignants, ce qui permettra de conférer plus de responsabilités et un meilleur contrôle de la gestion des soins aux patients. Dans ce document, nous réfléchissons à notre propre apprentissage en tant que communauté de professionnels de la santé issus de différentes disciplines. Nous reconnaissons l'importance croissante de l'implication du patient pour stimuler l'évolution des soins de santé, et imaginons un concept de centre de santé numérique et personnalisé via site Web pour la prise en charge intégrée des patients. Grâce à la collaboration entre patients, professionnels de la santé et structures pédagogiques, nous partageons nos premières expériences en matière de mise en œuvre d'un centre de santé regroupant des patients présentant des fractures de la hanche. Nous dévoilons également notre vision d'avenir pour les soins de santé, qui repose sur la cocréation de centres de santé numériques adaptés aussi bien aux besoins des patients qu'à ceux des soignants. Ce concept pourrait faire progresser l'enseignement et la pratique pour les professionnels du secteur, mais aussi améliorer leur efficacité; favoriser la participation des patients et soignants dans les soins auto-administrés; et enfin, permettre la collecte des données fournies par les patients, et nécessaires à la poursuite des recherches et améliorations dans le domaine des soins de santé.

## Resumen

La multimorbilidad es la presencia de más de una enfermedad crónica en un individuo. Los factores medioambientales, culturales, socioeconómicos y los relacionados con la salud, así como el comportamiento de los pacientes, influyen en los resultados de la multimorbilidad. Por lo tanto, se requiere un cambio en el tratamiento desde el modelo de daño físico hacia una atención integrada y centrada en el individuo con una mayor participación del paciente para abordar estos factores biopsicosociales complejos y a menudo interactivos. La educación influye en el comportamiento y se puede utilizar para que los pacientes y sus cuidadores tengan más capacidad de acción, lo que permite una mayor responsabilidad y control sobre la gestión de la atención al paciente. En este documento reflexionamos sobre nuestro propio aprendizaje como comunidad de profesionales de la salud de diferentes disciplinas. Se describe el concepto de un centro de salud virtual personalizado para la atención integrada del paciente, al reconocer la creciente relevancia de la participación y la acción del paciente en el proceso de evolución de la atención médica. Gracias a la colaboración entre las comunidades de pacientes, de salud y de educación, compartimos nuestra experiencia inicial sobre el establecimiento de un centro de salud en torno a una cohorte de pacientes con fracturas de cadera. Asimismo, describimos una visión de la futura atención médica basada en la creación conjunta de centros de salud virtuales que se centran en las necesidades de los pacientes y de los cuidadores. El centro de salud permitiría alcanzar importantes avances y mejoras en la práctica y la educación de la fuerza de trabajo; en el compromiso de los pacientes y los cuidadores con el autocuidado de la salud; y en la recopilación de los datos sobre la salud que los pacientes comunican y que se requieren para la investigación y las mejoras continuas en la atención médica.

## ملخص

تعدد الأمراض هو وجود أكثر من حالة مرضية مزمنة واحدة لدى الفرد. تؤثر العوامل الاجتماعية الاقتصادية، والعوامل الثقافية والبيئية، المرتبطة بالصحة، وكذلك سلوك المريض، على نتائج تعدد الأمراض. إن التعامل مع هذه العوامل البيولوجية النفسية الاجتماعية المعقدة، والمتداخلة غالباً، تتطلب تحولاً في العلاج من نموذج الضرر الجسدي إلى الرعاية المتكاملة التي تركز على الشخص، معتمكين أكبر للمريض للمشاركة وتحمل المسؤولية في العلاج. يؤثر التعليم على السلوك، ويمكن استخدامه لتمكين المرضى ومقدمي الرعاية لهم من خلال ومشاركة أكبر، وبالتالي السماح بمزيد من المسؤولية تجاه إدارة رعاية المرضى، والتحكم فيها. نركز في هذه الورقة على تعلمنا كمجتمع من الممارسين الصحيين من مختلف التخصصات. في ظل إدراك الأهمية المتزايدة لتمكين المريض في دفع تطور الرعاية الصحية، فإننا نصف مركز الصحة الرقمية الشخصي على شبكة الإنترنت للرعاية المتكاملة للمرضى. وبناءً على التعاون بين مجتمعات المرضى والصحة والتعليم، فإننا نشارك تجربتنا المبكرة في تنفيذ مركز صحي حول مجموعة من المرضى الذين يعانون من كسور الورك. كما نصف أيضاً رؤية للرعاية الصحية المستقبلية استنادًا إلى الإنشاء المشترك لمراكز الصحة الرقمية التي تركز على احتياجات المرضى ومقدمي الرعاية. يمكن أن يسمح المركز الصحي بتحقيق تطورات وكفاءات هامة في ممارسة القوى العاملة والتعليم؛ ومشاركة المريض ومقدم الرعاية في الرعاية الذاتية؛ وجمع البيانات الصحية التي قدمها المريض، والمطلوبة للبحث والتحسينات المستمرة في الرعاية الصحية.

## 摘要

多重病症是指一个人患有一种以上的慢性疾病。与健康有关的因素，社会经济、文化和环境方面的因素以及患者行为都会影响多重病症患者的治疗结果。因此，若要解决这些复杂且经常相互作用的生物心理社会因素，需要将治疗从物理性损伤模式转换为以患者为中心的综合护理，并提高患者在其中所发挥的作用。教育影响行为并且可以用来为患者及其照顾者赋权，让他们发挥更大的作用，从而对患者护理的管理工作承担更大的责任和管控力度。在本文中，作为一支汇聚不同学科从业人员的团队，我们反思了我们总结的经验。我们意识到患者在推动医疗护理改革方面所发挥的作用日益重要，因此我们描述了一个基于网络的个人数字医疗中枢的概念，用于整合患者护理。通过患者、卫生和教育团体之间的合作互通，我们围绕一批髋部骨折患者分享了我们在医疗中枢实施方面的早期体验。我们还基于共建以患者和护理人员需求为中心的数字医疗中枢，表达了对未来医疗卫生事业的美好愿景。医疗中枢可以在人员实践和教育方面实现重要进展和效率提升；可以让患者和照顾者参与自我护理；可以收集患者自述的医疗数据，所收集的数据用于医疗护理事业的长期研究和提升。

## Резюме

Мультиморбидность это наличие у одного человека нескольких хронических заболеваний. Исход мультиморбидности зависит от факторов, связанных со здоровьем, социально-экономических, культурных и экологических факторов, а также поведения пациента. Таким образом, решение вопросов относительно этих сложных и часто взаимосвязанных биопсихосоциальных факторов, требует перехода в лечении от модели физического повреждения к комплексному медицинскому обслуживанию, ориентированному на человека и предоставляющему большую свободу действий пациенту. Обучение влияет на поведение и может использоваться для расширения возможностей пациентов и ухаживающих за ними лиц, предоставляя им больше ответственности и контроля за процессом лечения и ухода. В этой статье авторы рассматривают процесс собственного обучения как сообщества практикующих специалистов из разных дисциплин. Признавая растущую важность свободы воли пациентов как движущей силы эволюции здравоохранения, авторы описывают концепцию сетевого персонального цифрового центра здоровья для комплексного подхода к лечению пациентов. Опираясь на опыт сотрудничества между пациентами и сообществами здравоохранения и образования, авторы делятся первоначальным опытом в части создания центра здоровья для контингента пациентов с переломами шейки бедра. В статье также описана концепция здравоохранения будущего, основанная на совместном создании цифровых центров здоровья, сосредоточенных вокруг потребностей пациентов и лиц, осуществляющих уход. Центр здоровья может позволить добиться значительных успехов и эффективности в сфере практической деятельности и обучения медицинских работников, вовлечения пациентов и ухаживающих за ними лиц в процесс самопомощи, а также сбора предоставляемых самим пациентом данных о его здоровье, которые необходимы для текущих исследований и совершенствования системы здравоохранения.

## Introduction

Multimorbidity is the presence of more than one chronic disease condition in an individual. By viewing multimorbidity as a person-centred concept we acknowledge that the impact of a condition is influenced not only by health-related characteristics but also by socioeconomic, cultural and environmental factors, as well as patient behaviour.^1^^–^^3^ Addressing these complex and often interacting biopsychosocial factors therefore requires a shift in treatment for multimorbidities from a physical damage model towards person-centred integrated care with increased patient agency. Such a model allows patients to have greater responsibility for and control over the management of their care.

Complex models of care that involve multiple health and social care disciplines are increasingly being developed. A trial of integrated person-centred care for multimorbidity found that patients expressed overall satisfaction with care services although they did not gain significant improvements to their quality of life.^4^ Similarly, there is increasing evidence around the value of innovations in digital health applications and in the health workforce to improve efficiencies and quality of care, as driven by the needs of the local context. However, the driving forces for scaling-up these initiatives will be political and economic and involve health-care professionals and patient advocates.^5^^,^^6^ In this paper we examine our own learning through our combined perspectives as a community of practitioners from different disciplines. Further, we highlight the importance of patient agency in driving the evolution of health services that are empowered by improved, digitally enabled strategies for patient education. 

## Community-driven progress

As best practice in health care and the learning process within medicine has evolved, so have community attitudes towards health care. Historically, progress in medicine has been shaped primarily by the health workforce driving continuous improvements in health care. However, there is now recognition that greater access to health information has allowed the involvement of patients and their carers (both formal and informal) to be considered as part of a community of practice, which is also influencing ways of delivering health-care services.^5^ Moreover, increasing access to digital technology could result in further patient and community empowerment and influence the balance between vertical (institutional) and horizontal (community) governance systems. This synergy between patient desires, digital technology and health-care expertise could provide innovative solutions and change the direction in which health care evolves.^5,7^

## Patient education

The World Health Organization (WHO) *Global strategy on human resources for health: workforce 2030* report clearly outlines the challenge to providing universal health coverage with a projected deficit of 18 million health-care workers.^7^ A contribution to addressing the deficit could be through training a workforce for a defined scope of practice, supported by technology-assisted service delivery to better engage and empower patients and their communities. The workforce could help facilitate the collection and use of the immense amount of data (so-called big data) that can be captured from patients and could be involved in applying emerging artificial intelligence solutions to health care.^1^^,^^6^^,^^7^ Recently WHO introduced digital health as a broad term to encompass health services provided electronically (eHealth), including mobile health technology solutions (mHealth), as well as emerging areas, such as the advanced use of computing sciences to manage big data, genomics and artificial intelligence systems.^8^^,^^9^ A major challenge now is how to process digitally collected data and interpret it in a meaningful way.^2^^,^^9^ For health practitioners and patients alike, the amount of health information from sources, such as friends and family, the internet, medical journals, health pamphlets and specialists can be overwhelming and lead to confusion rather than clarity. Differing levels of health literacy already affect patients’ ability to understand health information and to make informed decisions about their health. Differences in ability to access and use technology, the so-called digital divide, create further inequities in access to health information.^1^

While electronic health records allow communication and data management among health-care providers, these types of records were not designed with the primary goal of engagement with patients. Some software applications on mobile devices are designed to collect data for use by health professionals, such as patient-reported outcome measures. Yet the information flow usually offers limited, if any, effective ways to involve patients in their own care. Overall, there has been a rapid increase in the availability of mHealth applications,^10^ by a variety of vendors including health agencies, fitness advocates and software companies.^11^ These health applications are primarily designed to support patients or consumers^12^ in the domain of general lifestyle and wellness, such as applications on mobile phones or wearable devices that monitor activity levels or heart rate. However, there are also developments in the use of eHealth services to support the management of specific diseases (such as rheumatoid arthritis, diabetes, anxiety and mood disorders) in conjunction with specialist services.^1^^,^^12^ For example, there are now applications for patients with diabetes, which can be synced to small monitoring devices inserted subcutaneously to monitor glucose levels continuously. This technique allows controlled insulin delivery in wearable automated pumps while providing critical feedback to both patients and their carers to inform necessary medication, dietary and lifestyle adjustments. Some of these applications have readily become accessible and inexpensive, reducing the need for expensive doctor visits and laboratory-based investigations.^12^ However, access to such technologies is not universal for patients or health-care providers. Research can identify the technologies and processes that are most feasible for supporting effective implementation of digital health solutions. Applying evidence-based guidelines is therefore important for mitigating potential digital divides.^5^ There is likely to be a strong emphasis on the use of widely used technologies, such as mobile phones where patients and health-care workers are likely to have greater access to the technology.^13^ Applying best-practice guidelines at the inception of a new system will ensure coherence between traditionally established care practice and emerging, digitally enabled models of care.^5^^,^^9^

The success of some of these software applications in actually changing patients’ behaviour^11^^,^^14^ should come as no surprise if the principles and theories on which they are based are clearly understood.^15^^,^^16^ Information is provided to users in a format that is easily understood and meaningful in the context of users’ goals. The knowledge gained can be applied to solve users’ problems and the success of that application is assessed with immediate and specific feedback to the user. Based on this feedback the users are able to make the necessary changes required to further their individual goals and the cycle repeats. The theory is related to fundamental approaches to education, where the process of learning can bring about behavioural change. Most clinicians involved in faculty and student training are well aware of the principles. Yet how often are these principles applied as part of clinical practice? Unfortunately, simply providing information and awareness is not the same as providing education. Awareness and understanding of important information do not automatically improve an individual’s capability and translate into positive behavioural change.^1^ Patients, particularly those with chronic conditions, often do not adhere to treatment guidelines, such as prescribed medications, lifestyle changes, rehabilitation and exercise programmes. Given the many circumstances where patients mis-hear, misremember or misunderstand information and advice from their health professional, there will often be adherence issues. To translate knowledge into successful health outcomes, a co-designed and integrated approach to patient education is needed, with a consistent and shared understanding among all care providers.^17^ The same arguments that have led to increasing the agency of students in education can be applied to patient agency in health care.

## Personal digital health hub

A personal digital health hub can specifically collate and interpret useful health information, facilitating the integration of data from different health services and other personalized digital data sources. This personalized hub is potentially a powerful tool, empowering patients to take greater control of their health goals. The major limiting factor, however, is that current applications are not connected to mainstream health services and are not linked to the professional networks of family practitioners or specialists. In contrast, processes that are specifically designed around the individual can link patients into a more extensive network of specialists, general practitioners and community carers. This holistic approach is important for the development of a strongly integrated team approach that is more responsive to the needs of patients and communities. Models of care need to be further redesigned by deploying digital health solutions that will allow delivery of high-quality and patient-centred information to strengthen and integrate care closer to the community setting. There are existing eHealth applications used to enable the implementation of models of care in patients with arthritis^18^ and skeletal fragility.^19^^,^^20^ The applications streamline system-level referrals, build workforce management capacity and support patients in managing pain or performing exercises at home.^21^

Collection of selected data linked to patient-reported outcome measures would contribute to big data repositories, such as outcome registries and could be used for health research. The data might be used for validation of artificial intelligence-based predictive algorithms and decision-support tools and could inform improvements in the design of these tools.^1^^,^^5^^,^^17^ Blockchain technology, which provides a transparent, unalterable record of a transaction, offers a verifiable, permanent and attack-resistant method for recording health data. By increasing the security and patient trust in the quality and use of the data, a personal health hub can create opportunities to transform health care and place the patient at the centre of the health-care network.^5^ With these innovations, we envisage role shifts among health-care workers, in which diagnostic and prescriptive roles give way to more supportive, collaborative, nurturing and motivational skills to empower patients. Collectively, these technological innovations are expected to drive major changes in the composition, scope of practice and training (based on required capabilities) of the workforce, allowing more efficient use of resources to deliver the right care, at the right time, in the right place, by the right person with the right resources.^5^

In the absence of a strong sense of need and urgency, and to manage resistance to change in well entrenched care practices, this approach will need to be introduced in stages. Depending on the organizational model used within different health-care settings, implementation could be initiated either through empowered specialty groups or from within primary care. With linkages to the patient and community services established, new systems could be further optimized around a variety of chronic disease models of care. Eventually the systems could transition into a patient-controlled and government-supported system, linked to the mainstream suite of public and private health services. The personal digital health hub could connect with other wellness providers, such as nutritionists, physical therapists, psychologists and social workers; and a myriad of suitable, commercially available mHealth applications. Patients, whether living independently or under a care arrangement, could have their own customized health hub with the ability to share relevant and selected categories of information with health-care providers and community or social support networks. The shared platform could also function as a virtual workplace for vocational training, allowing a mutually transforming process of learning (both cognitive and sociocultural), through participatory practice^22^ of patients, carers, health-care practitioners and students alike. 

The personal digital health hub would be specifically built around patients. All stakeholders would have a role to play in supporting the individual’s health literacy. Care goals could be integrated, providing a more holistic approach to care with improved health outcomes and health-system efficiencies.^1^

Adaptable software solutions to create a digital hub already exist in the form of learning management systems that are widely used in the education sector.^5^ Many of these systems are based on open-source software, which would facilitate their future adaptation and implementation in low- and middle-income economies. The roles of course administrators, teachers, students and observers can be substituted by health-care providers, liaison officers, patients and family or carer supporters. These platforms have design features that provide services, such as secure internet access for users; content management; monitoring of the volume and frequency of communication exchanges between participants; progress tracking of assigned tasks; and assessment of users’ engagement through data on time spent online and on specific pages or tasks. Patient-reported outcome measures can be readily collected to monitor individual outcomes, as well as contribute de-identified data to large health research databases. Plug-ins allow the data and functionality of existing or new mobile applications to be integrated into the hub software and accessed through both web and application-based interfaces. A community of learners can be created to support the education of health-care workers and patients alike.^1^^,^^23^

Clearly, there are many challenges to be addressed. Governments would need to invest in this approach through both policy and funding, but the potential gains extend well beyond the health sector to actual improved national productivity.^6^ Cloud access (the ability to access files stored on internet servers), information storage and security using emerging blockchain technology would need to be addressed. However, there are policies and procedures within existing health and governance structures that can address issues, such as patient confidentiality and ownership of data, and could be adapted and implemented effectively. As a contained system the digital health hub could also ensure that the data mined remains fully transparent, patient-controlled and used solely for the purpose of analysing and influencing health behaviour and enhancing health-care outcomes. The system should ideally remain completely separate from control by data brokers with commercial and political interests. The technologies used to secure data would support this to some extent, but the use of open-source software and the local political environment will likely be influential.

## Early experiences

Our concept of a digital health hub has evolved since 2012 when we established a telephone-based remote follow-up and virtual clinic service for hip fracture patients. With 500–600 patients annually, the Royal Adelaide Hospital is one of the busiest hip fracture centres in Australia. In this cohort, we considered all patients as remote, even those living locally, due to the logistics of travel and support required to attend a hospital-based clinic.

To address the challenges and insights that emerged from the virtual clinic we designed a digital patient health hub using a transdisciplinary approach in this specialist orthogeriatric setting.^17^ Input was provided from clinical disciplines (geriatrics, orthopaedics, emergency medicine, anaesthesiology, rehabilitation medicine, general practice, nursing, allied health and pharmacy); non-clinical disciplines (health economics, computer science, higher education, mathematics, architecture and demography); and patient and consumer groups. We used a collaborative and co-design approach to translate our knowledge and experience into successful health outcomes.

The digital health hub was designed to improve education, service integration, data exchange and engagement of all stakeholders including patients and health-care providers.^17^ We structured the web-based platform to provide information related to health issues under four key sections: (i) current concerns (for example, a hip fracture); (ii) essential wellness (nutrition, exercise, sleep and mind); (iii) community health (hygiene, contagious diseases); and (iv) past health. The digital hub was thus designed to support a lifelong approach to healthy ageing through lifestyle approaches, while addressing injuries or illnesses as they arise. Preliminary background research of this elderly patient cohort and their carers confirmed that they had significant capacity to access digital health solutions through the support of networks of carers.^9^

As we develop this new model of care, nurses or other health-care workers with defined competencies in orthogeriatrics, would be further trained to fulfil the additional roles of a fracture liaison coordinator, an online educator and a facilitator of behavioural change. Patients and carers will be engaged from the time of admission and provided with instructions and secure access via the digital hub to resources designed to provide a clearer understanding of the complete course of hip fracture. A liaison officer coordinating the information exchange and engagement through the digital hub will be the first point of digital contact, while the patient or designated carer retains control of access rights for additional carers and observers. The liaison role will be further supported by decision-support protocols with oversight by, and ready access to, orthopaedic and geriatrician specialists.

We expect that a wide range of quality, evidence-based educational resources will be adopted, adapted or developed in partnership with patient and consumer groups and delivered through the online learning platform. The resources will be made available in a variety of suitable digital formats to address individual educational needs around understanding of the injury, management and support options (including surgery, anaesthesia, pain, thromboprophylaxis, discharge medications, nutrition, exercise and post-operative mobilization, sleep, wound care, falls risk assessment, osteoporosis, sarcopenia, frailty, cognition, advance directives and community services). These educational resources are similarly used to inform the associated community health-care professionals and students engaged with the patient in the digital health hub.

A calendar, with functionality for several reminder options, will be used to schedule and manage follow-up tasks, including progress feedback, appointments and community-based investigations. Further communications can be via email, text message, telephone, videoconference or face-to-face appointments, as required. Patient engagement is tracked using multiple metrics, which are monitored and captured, such as time spent in specific areas of the health hub, communication exchanges and tasks completed. Feedback of patient progress will be primarily digital, with telephone follow-up as required, and include information used to both inform immediate clinical management and provide patient-related outcome measures for audit and research purposes.

We believe that important progress towards a more patient-centred and integrated health-care system can be made through the collective wisdom of health-care providers from multiple disciplines in partnership with patients. Nevertheless, this new model of care and the digital health hub, while showing great promise in this challenging cohort of older patients with a hip fracture, are still in a development phase. Further refinements of the digital health hub will be informed by the iterative development process, gained from user feedback and analysis, before wider implementation and evaluation in the specialist setting. This will be followed by application of the model to manage conditions involving other specialty areas and ultimately by adaptation for use in a community-based primary-care setting.

## Other settings

For low- and middle-income countries, the use of open-source software and mobile phone technologies may provide the greatest opportunity to support universal health coverage through contextualizing a personal digital health hub. Mobile phone penetration is high in many low- and middle-income countries and mHealth is already recognized as promising to provide patient-centred care in some of these countries.^24^ Emerging evidence reflects digital health being used in these settings to strengthen primary health-care systems^25^ by targeting service delivery and increasing community health workforce capacity;^26^ improving health education and lifestyle behaviours;^27^ and supporting self-management of noncommunicable diseases.^28^ Some digital health interventions that were focused on capacity-building or training of community health workers were not informed by the theories in education and, ironically, lacked an understanding of what counts as learning.^29^ We expect that a system built on best-evidence education principles would be more likely to succeed.

## Future directions

We envisage a world where person-centred, integrated health care is provided holistically; the patient is an active and health-literate partner; and health-care workers act as life coaches, competent in the principles of online education and behaviour modification. Multimorbidity will be managed more efficiently in the community, aided by digital personal health hubs linked to best-practice content most relevant to the context. ^1^^,^^17^
